# Exploring the Experiences of Individuals With Type 2 Diabetes in the Context of Alcohol Use and Lifestyle Change: A Phenomenological Study

**DOI:** 10.1111/scs.70149

**Published:** 2025-11-14

**Authors:** Christine Mantzouka

**Affiliations:** ^1^ School of Health and Community Studies Leeds Beckett University Leeds UK

**Keywords:** alcohol consumption, lifestyle changes, type 2 diabetes, well‐being

## Abstract

**Background:**

Individuals with type 2 diabetes who consume alcohol require lifestyle changes, including altering food and alcohol consumption. Alcohol and food consumption for individuals with type 2 diabetes create an accumulative impact that adversely affects their potential to alter their lifestyle.

**Aim:**

This study explores how individuals with type 2 diabetes who consume alcohol at varying levels interpret and manage lifestyle changes related to drinking and diet.

**Methods:**

The study uses hermeneutic phenomenology. Semi‐structured face‐to‐face interviews were conducted with 12 participants living with type 2 diabetes who consumed alcohol. Data were collected from September 2017 to February 2018. The University's ethics committee approved the study, and the data were analysed using thematic analysis.

**Ethics:**

Ethics committee approval was obtained from the University of Leeds Committee on Research Ethics, and written informed consent was obtained from participants.

**Findings:**

Lifestyle modifications for individuals with type 2 diabetes who consume alcohol are more complex than for individuals with type 2 diabetes who do not. Also, the impersonal nature of organisational and professional culture impedes lifestyle changes. Furthermore, insufficient, unfriendly and incomprehensible labelling of food and alcohol impedes healthier food and alcohol selection choices. Finally, family, friends, and other social networks unintentionally encourage alcohol drinking and food consumption.

**Conclusions:**

The increased number of group memberships or the potential to develop new group memberships increases the likelihood of commencing and sustaining lifestyle changes. Also, healthcare professionals need to educate individuals with type 2 diabetes who consume alcohol to read, evaluate and interpret food and alcohol labels, and act upon policies regulating the food and alcohol industry. Health professionals need to empower individuals living with type 2 diabetes to develop new social group memberships. Furthermore, an integral component of the caring process is the co‐construction of explanatory, sense‐making and transformative narratives that justify and warrant lifestyle changes. Finally, health professionals need to re‐envisage their healthcare roles and reshape how they employ information technology and integrate family in the lifestyle changes.

## 1 Introduction

The global health challenge of Type 2 diabetes (T2D) continues to expand rapidly since it currently affects over 537 million adults worldwide and projections suggest this figure will increase to 643 million by 2030 according to the International Diabetes Federation (IDF) Diabetes Atlas 2021 [1]. People affected by T2D experience high blood sugar levels because their bodies either resist insulin or produce insufficient amounts of it leading to serious long‐term health problems such as cardiovascular disease alongside kidney damage and loss of vision (American Diabetes Association [ADA], [2, 3]).

Sustained lifestyle modifications together with dietary changes and physical activity play a key role in T2D management and such individuals also need to reduce or regulate their alcohol intake according to ADA guidelines [2]. The practice of alcohol consumption remains deeply embedded in society's traditions and carries significant emotional depth which links to social connection, personal identity formation, and relaxation techniques. Alcohol consumption presents a critical behavioural and psychosocial issue for T2D patients because it disrupts glycaemic control and affects both medication adherence and weight management [4].

Despite increasing recognition of the importance of behaviour change in T2D care, many healthcare systems and clinical interactions remain overly focused on biomedical targets, often overlooking the lived realities that shape patients' abilities to initiate and sustain lifestyle change [5, 6, 7]. This study focuses specifically on individuals with T2D who consume alcohol at varying levels, seeking to understand how they experience and make sense of lifestyle change in the context of their everyday social, emotional, and cultural environments.

## 2 Background

In the UK and Europe people commonly consume alcohol which serves the social functions of relaxation, celebration and fostering group belonging [8, 9]. People diagnosed with T2D face distinctive physiological and behavioral challenges when consuming alcohol. Moderate alcohol consumption leads to impaired insulin sensitivity, disrupted blood glucose regulation and weight gain which makes disease management more complex and increases complication risks [2, 10]. Whilst total abstinence from alcohol is not necessary for everyone, nevertheless people with T2D who reduce their alcohol consumption may see benefits [2].

The significance of alcohol consumption among T2D individuals involves more than just physical health repercussions. Social norms together with individual identity profoundly shape alcohol consumption habits. Drinking helps numerous people retain their social connections, manage stress levels, and maintain normalcy during chronic illness periods [11, 12]. This population requires lifestyle changes that extend past behaviour modification to include existential and relational dimensions. However, it is essential to avoid reinforcing diabetes stigma by presenting T2D solely as a lifestyle condition, as genetic and other non‐modifiable factors also contribute to its onset [4].

People often view attempts to cut back on alcohol consumption or alter their dietary habits as actions that lead to social alienation and emotional difficulty or threaten their sense of identity. Social expectations of the immediate environments and support systems may reinforce T2D individuals' sense of fear, denial, and resistance to reducing alcohol consumption [13, 14]. Health professionals who rely exclusively on technical advice without attending to these lived realities may inadvertently and further reinforce resistance or stigma [4, 15].

Lifestyle change, in this context, must be understood through the lens of lived experience. A phenomenological approach which focuses on the lifeworld of individuals offers a valuable way to explore how people with T2D experience the tensions between lifestyle advice, social belonging, and personal meaning [16, 17]. In this study, lifestyle changes are defined not only as measurable behavioural adaptations (e.g., reduced alcohol use, changes in diet), but also as shifts in self‐perception, social participation, and meaning‐making processes [18, 19].

## 3 Aim

This study explores how individuals with T2D who consume alcohol at varying levels interpret and manage lifestyle changes concerning their alcohol use and diet within the social, healthcare, and cultural contexts of their lives.

## 4 Methods

### 4.1 Study Design

This study employs a hermeneutic phenomenological methodology inspired by Van Manen's [17, 20] and Dahlberg and Dahlberg's [21] writings. Although Dahlberg and Dahlberg's emphasis on openness and bridling informed the overall posture toward the data [22], the analytic stance remained interpretive, aligning with Van Manen's view of research as a process of meaning‐making through dialogue between the text, context, and researcher. In a nutshell, hermeneutic phenomenology recognizes that human experiences are inherently interpretive and contextually shaped by the fusion of the participant's lived world and the researcher's own perspectives.

The researcher maintained reflexivity throughout the research by keeping a reflective journal and memo notes throughout all stages of data collection to examine their assumptions, values, professional background, and emotional reactions to participant stories [23, 24, 25]. Reflective practice enabled the researcher to maintain self‐awareness of the interpretive influences while working toward openness and sensitivity to participants' perspectives. The researcher had no prior relationship with the participants, which helped establish trust and support the credibility and authenticity of the interpretations. To support the trustworthiness and transparency of the study, this qualitative research follows the COREQ (Consolidated Criteria for Reporting Qualitative Research) guidelines.

### 4.2 Setting

The study was conducted within the context of diabetes support groups in the UK, specifically in the North of England. The researcher engaged with five different support groups to approach and conduct interviews. These interviews were held either in the regular meeting venues of the support groups, private rooms in local libraries, or at the University's premises. This setting provided a familiar and comfortable environment for the participants, facilitating open and honest discussions.

### 4.3 Recruitment and Data Collection

Twelve individuals diagnosed with T2D for at least 6 months, and consuming varying levels of alcohol were purposively selected to participate in the study. The 6‐month threshold was chosen to ensure that participants had moved beyond the immediate post‐diagnosis period, during which national guidelines recommend the provision of structured education and support [26, 27]. This timeframe was intended to ensure participants had received initial information and had the opportunity to begin managing their lifestyle in response to their diagnosis.

Participants were recruited from five diabetes support groups located in two UK regions. The researcher approached group facilitators and introduced the study during in‐person visits and via email correspondence. During visits, the researcher gave a short verbal introduction to the study at the beginning or end of the group sessions and distributed information sheets. Interested individuals were invited to contact the researcher privately, either during the session or via email at a later time. Recruitment conversations were conducted one‐to‐one in private, without the involvement of group facilitators or other members, to maintain confidentiality and ensure voluntary participation.

Alcohol consumption was categorised following Koloverou et al. [28] into low (> 0 but < 1 glass/day), moderate (1–2 glasses/day), and high (more than 2 glasses/day). While alcohol dependence was not the focus, participants' reflections often revealed how food and alcohol consumption intersected in shaping their capacity for lifestyle change.

The study, following the qualitative principles of sampling, used non‐probability sampling techniques, ensuring the representation of the phenomenon rather than the representation of the population [29]. Morse [30, 31] asserts that it is not only counterproductive but may adversely impact the qualitative study's validity if the qualitative researcher focuses on the statistical representation of the population's demographics rather than deliberately selecting those participants who have the experiences and are willing to share them. Moreover, the qualitative sample is small in numbers by its nature, and attempting to achieve a statistical demographic representation of the sample is problematic and unhelpful as such small numbers cannot lay claims to any genuine representation of the total population [32]. Consistent with this literature the participants of this study were intentionally selected based on the wealth of their experience and their willingness to share this.

Data were collected through 12 one‐to‐one, semi‐structured interviews between September 2017 and February 2018. Interview questions were informed by the literature on identity in chronic illness [33], diabetes‐related stigma [34], and phenomenological concepts of the lifeworld [17, 22]. The guide focused on how participants perceived and managed the relationship between diabetes, alcohol, social belonging, and lifestyle change. Participants consumed alcohol at varying levels, from occasional to regular intake, but none were diagnosed with alcohol dependence.

The interview schedule was piloted with two individuals who met the inclusion criteria. Their feedback on question clarity and emotional tone informed minor revisions to wording and structure. These pilot interviews were included in the final dataset, as their depth and relevance matched those of the subsequent interviews.

Each interview lasted between 40 and 70 min and was conducted in a location of the participant's choosing (e.g., private room in a community centre, or a neutral public venue). All interviews were audio‐recorded with consent and supported by field notes to capture non‐verbal cues and contextual details. Interviews continued until data saturation was achieved, defined as the point at which no new themes or insights emerged from the final interviews. The final interview guide is provided as Appendix A to ensure transparency and support reproducibility.

### 4.4 Data Analysis

To analyse the data, Van Manen's heuristic guide of analysing phenomenological data, Clarke and Braun's reflexive thematic analysis framework, and Saldana's step‐by‐step practical coding and thematisation process were used as described in Table 1 ([35], 591–593, [36, 37], 5; [17], 212–230). The first column summarises Van Manen's heuristic guide to phenomenological analysis. The second column outlines Clarke and Braun's thematic analysis framework. The third column draws on Saldana's coding and thematising steps. The green column highlights coding stages. An example of the coding and theme generation example is provided in Appendix B.

**TABLE 1:** Integrated framework for hermeneutic phenomenological data analysis.

Van Manen's heuristic guide	Clarke and Braun's thematic analysis framework	Coding/categorising and thematising steps
	Familiarisation with the data: reading and re‐reading the data	
Uncovering thematic aspects in lifeworld descriptions: thematic phrase pointing at an aspect of the phenomenon	Coding: generating pithy labels for important features of the data of relevance to the research question guiding the analysis	In Vivo Coding—1st cycle: a label that refers to a word or short phrase from the actual language found in the raw data
Isolating thematic statements: read line‐by‐line asking each thematic phrase “*what does this sentence or statement reveal about the experience described?*”		In Vivo Coding – 2nd cycle: interpretation of codes from the 1st cycle by asking the initial code “*what does it mean, what is happening, or what are the assumptions?*”
Composing linguistic transformations: thematic statements from various sources are captured or put together	Searching for themes: coding your codes to identify similarities in the data	Subthemes: reflectively and creatively read and re‐read the codes to identify similarities, cluster them together, and develop taxonomies to determine “w*hat is in the data?*”
Gleaning thematic descriptions from artistic sources: reflective exercise that transcends the experiences	Reviewing themes: reflecting on whether the themes tell a convincing and compelling story about the data and define the nature of each theme and their relationships	
Determining essential themes: generating deeper insights into experiences	Defining and naming themes: identifying the “essence” of each theme and constructing a concise, informative name	Themes: categories are compared to identify relationships and develop highly abstract and meaningful essences that run through the data
Phenomenological writing: writing in sensitive and subtle undertones of language and allowing the text to speak for the experience	Writing‐up: weaving analytic narrative and vivid extracts to tell a persuasive story situated in the literature	

### 4.5 Ethical Considerations

Ethical approval for the study was obtained from the University of Leeds Ethics Committee (SHREC) (Ethics Reference: SHREC/RP/527). Participants were provided with an information sheet and invitation letter, followed by oral clarifications. Informed consent was obtained before data collection. All data were anonymised, and participants' details remained confidential, stored on a password‐protected server accessible only to the researcher. Participation was voluntary, with the option to withdraw at any stage without consequences. The study posed minimal risk to participants and researchers, with no questions expected to cause stress or anxiety.

### 4.6 Findings

The analysis generated three major themes; each composed of subthemes. These themes reflect the lived experiences of individuals with T2D who consume alcohol at varying levels, particularly as they navigate the complexities of lifestyle changes in relation to social identity, healthcare systems, and cultural contexts.

In line with Braun and Clarke's [36, 38] reflexive thematic analysis approach, themes are conceptualised as overarching patterns of meaning across the dataset, while sub‐themes represent distinct yet interconnected facets that support each theme. The findings illustrate the relationships between themes and sub‐themes, as well as their connection to the research question.

### 4.7 Theme 1: Influences of Collective Determinants on Lifestyle Changes

Social systems, including group memberships, food and alcohol culture, the consumer industry, and online media, significantly influence the lifestyles of individuals with T2D who consume alcohol. These systems can either facilitate, or hinder lifestyle changes related to food and alcohol consumption.

#### 4.7.1 Subtheme 1. Group Memberships and Identity Construction

Participants often perceive social group membership as intertwined with activities such as pub‐going, alcohol consumption, and eating out. Such social practices can limit individuals' control over their eating and drinking habits, creating a conflict between the need to manage T2D and the norms of social gatherings. Participants who sought out social groups that de‐emphasized alcohol and food consumption willingly accepted changes in their social identities.

I used to go to the pub almost every night…I knew the owner of the pub. I mean I knew most of the people in there … [and] I like whiskey, so I used to have at least one [glass] whiskey every night, maybe two or three if others joined and started buying rounds or doing shouts that you have to keep up with… I feel that I need to stay home to control what I drink and eat [but] I miss the days before [my diagnosis with diabetes] that I could join family and friends for a meal or drink. (P6)

#### 4.7.2 Subtheme 2. Marketing Practices of the Consumer Industry

The following participant highlighted the inadequacy of the nutritional labelling system on foods and drinks, which they found either lacking or incomprehensible. A standardised, comprehensible labelling system could significantly aid individuals in making healthier choices. For participants who consumed alcohol at varying levels, nutritional labels on both food and beverages were often confusing or incomplete. While this challenge is not exclusive to these individuals, participants described how unclear labelling on alcoholic drinks compounded their difficulty in making informed choices and controlling blood sugar.

Τhey don't have labels on them, stuff like different types of bread that can make a big difference and cakes can make a difference. But I do feel that these food manufacturers can help a lot more… (in cases they provide nutritional information) it's over the top unless you're in the profession you wouldn't understand it. I feel that rather than being voluntary, it should be compulsory. (P1)

Pharmaceutical companies also play a role in self‐managing T2D by providing supplies and financial reimbursement, which improves glycaemic control. However, easy prescription or over‐prescription of medication can act as a deterrent for individuals with T2D who consume alcohol at varying levels to strive for lifestyle changes and over‐rely on medication for controlling their T2D condition.

Some major pharmacy companies will help you with diet and blood testing and that way. My GP sometimes prescribed some free test stripes, but they are very limited, so I don't get many. I originally started on a diet but moved on to tablets, and now gradually increasing the number of now tablets. Well, I try and exercise. I don't do enough, so I take my tablets. (P9)

#### 4.7.3 Subtheme 3. Health Literacy and Lifestyle Changes

Participants' experiences with online information varied. While some found it overwhelming and misleading, others benefited from the immediacy and practical advice offered by online social networks.

Going to the internet and finding information was a grim experience because you read about amputations, blindness, kidney disease, all the horrors you can get from it, and in one of the wards…Then if you compare it with what's in the media and what's coming out from some online sources, it's sometimes difficult to be clear what is the best way to deal with diabetes. (P2)

In summation of theme 1, for many participants, food and alcohol consumption held symbolic meanings and shaped their social identities. Lifestyle modifications for these individuals are complex and intertwined with their social networks. Multiple group memberships can facilitate the construction of new social identities, promoting lasting changes in food and alcohol consumption.

### 4.8 Theme 2: Contextual and Cultural Framing of Lifestyle Changes

The healthcare system's organisational structure and the interactions within it significantly influence the experiences of individuals with T2D who consume alcohol at varying levels and their efforts to alter their lifestyles.

#### 4.8.1 Subtheme 1. Interpersonal Approaches of the NHS

Participants reported formulaic and impersonal interactions with NHS healthcare professionals, which focused on dietary consumption and weight targets without addressing underlying issues. Furthermore, the participants felt their interactions with health professionals were routinized, lacking any personal involvement in the deliberations about treatment options or considering their potential to understand the provided information, ergo transforming the care provision into an upsetting process obscuring individual needs. They also noted a lack of information on alcohol consumption.

They (healthcare professionals) didn't provide me with any information about alcohol or lifestyle… The only thing she (nurse) would say was, you have to take your weight down, but nothing on how I can do that. The doctors and the nurse didn't give me enough information. I went to the hospital at (name of the city), and they just gave me this about diet, and I thought, what is this? What do I do? (P10)

Participants also felt that NHS financial cutbacks and structural changes negatively impacted the support they received for managing T2D. The changing nature of the NHS, the absence of early diagnosis mechanisms, and the continuous reforming of NHS structures subtly pass on the responsibility to individuals to do scanning tests, rather than these being part of the NHS prevention policies and the need for individuals to seek care outside the NHS structures.

#### 4.8.2 Subtheme 2. The Interactional Nature of Support Groups

Support groups were viewed as open, interactive forums that provided a sense of belonging, intimacy, and a safe space for sharing personal experiences. They served as collective buffers for emotional distress and facilitated the construction of new life stories. Finally, the participants conceive support groups as the places where individuals gain a sense of control in accessing information, resources and support independent of established and authoritative professionals.

The only place I have learned about diet is here (diabetes support group). And this group helped me, it is good. And it helps me in other aspects … I'm hoping to get a more social thing here… I think if we can have a good Christmas party next week, we meet once a month and we have had a lot of new people just hoping that we can keep them there is a social side of the group, and this helps, well in my experience it does help, and people look forward to coming here. Here I know I am not alone, and I know others, like me, feel not sure what the future has in for us. (P1)

#### 4.8.3 Subtheme 3. The Professional Culture of the NHS

Participants sensed that limited interaction with healthcare professionals led to negative labelling and insufficient information exchange, hindering lifestyle changes. Conversely, positive interactions with healthcare professionals allow exchanging information and re‐focusing life narratives toward developing a hopeful future.

He (doctor) was very helpful because the first thing he said to me was it's not your fault and I think that was so important and we had a very general discussion about lifestyle, about portion sizes and watching what I ate… I'm very pleased with the Health Professionals, and I see a GP at the (name of medical centre), and again he was very supportive in terms of giving me the diagnosis and reassuring me and putting it in a different view. (P3)

In summary of theme 2, the NHS organisational and professional culture often emphasises hierarchical structures and impersonal interactions, impeding lifestyle changes. However, positive and interactive engagements with healthcare professionals can significantly support individuals with T2D in managing their condition.

### 4.9 Theme 3: The Embodiment of Intersubjective Interactions

Interactions with family and friends play a crucial role in facilitating or hindering the meaning‐making process for lifestyle changes and decision‐making for individuals with T2D who consume alcohol at varying levels.

#### 4.9.1 Subtheme 1. The Impact of Individuals' Networks on Lifestyle Changes

Supportive family environments, where dietary changes are made collectively, can reinforce the commitment to lifestyle changes and symbolize family cohesion. On the contrary, unsupportive behaviors and misconceptions within the family can create a non‐conducive environment for change.

You know I've cut down on food like chips and that sort of stuff. We've done it as a family really, rather than just me as an individual. At first, it was a bit difficult because all of us as a family I have two teenage boys in college, and they want to eat something that I can't, but very soon I got them in my way of eating and way of thinking, and now they enjoy the food I eat. (P7)

#### 4.9.2 Subtheme 2. External and Internal Factors Impacting Lifestyle Changes

Participants who found meaning in the change process and could relate it to past successful health experiences were more likely to integrate the required changes into their lives.

I like reading things. I have had to because of my other problem, my neuromuscular disease. So from experience I have had for learning about that condition, I think it set me up quite nicely to pick up on what was good with diabetes and what wasn't good with diabetes. (P12)

However, individuals who cannot find meaning in the change process or are unwilling to accept the chronicity of their condition out of fear of altering their identity as healthy individuals are usually unable to achieve and sustain lifestyle changes. A further obstacle to lifestyle changes is others who may either not believe the individual has a health issue as there are no immediate signs of diabetes or consider the individual morally responsible for being of weak character and therefore developing diabetes.

When diagnosed, I went onto medication. I found that quite depressing because I thought of a pretty fit person, all of a sudden, and you go on medication for life. I did find it hard to cope. When first diagnosed, they said, ‘Oh, we need to put you on medication’. I did fight against it because I don't know if I thought it was a sign of weakness. I found this change over quite hard… I'm not sure if some people actually think, well it's his fault he's been overweight for so long he's now got his rewards. (P7)

#### 4.9.3 Subtheme 3. The Embodied Significance of Food and Alcohol Consumption

Participants considered food and alcohol consumption as reflective of their mood and feelings, serving as mechanisms for relaxation and social interaction. These activities helped construct social identities and foster a sense of belonging. Lastly, food consumption and alcohol drinking act as unwinding mechanisms, and individuals tend to use them as adaptive responses to life events.

I was comfort eating when Paul (husband) died, plus drinking a little bit while I shouldn't be drinking. I believe there is a lot of truth in comfort eating. But if you direct yourself to something else (instead of eating), you forget about eating because you are engrossed in something else… It's crept up a bit since I've been out of work (alcohol drinking). You're so cheesed off being out of work; It's stressful thinking when I am going to find a job, and alcohol helps me relax and not think all this. (P10)

In summary of theme 3, personal histories, the mental capacity to consciously make choices, and the ability to provide meaning to lifestyle changes are significantly influenced by daily interactions with family, friends, and social networks.

## 5 Discussion

This study explored how individuals with T2D who consume alcohol at varying levels interpret, experience, and respond to lifestyle change within the context of their social identities, healthcare interactions, and cultural contexts. By using a phenomenological lens, the discussion highlights the meaning‐making processes that underpin participants' choices and challenges in sustaining lifestyle modifications.

A key finding of the study is the central role of food and alcohol in shaping participants' social identities and collective belonging. For many, alcohol consumption and communal eating were not merely behaviors, but practices embedded in social rituals and group norms. As such, lifestyle change represented not just a medical adjustment but an identity negotiation requiring individuals to redefine how they belong to and are recognized within their social worlds [39, 40]. This echoes previous research on identity conflict in chronic illness [33, 41] and affirms the need for interventions that support identity reconstruction during behavior change.

Participants who sustained behavioural change achieved this through the creation of new social connections or by redefining their roles within existing groups. The Social Identity Model of Behaviour Change by Haslam and his colleagues [42] establishes that sustained behavioural change relies on social connection and the support provided by group affiliations. When participants developed new identities through walking clubs or diabetes groups their lifestyle adjustments aligned with their new self‐conception which demonstrates how change is relational and context‐dependent [43, 44].

Structural and informational barriers further complicated participants' efforts to engage in lifestyle changes. Participants voiced frustration with food and alcohol labeling that was ambiguous or overly technical echoing concerns raised in public health literature [45, 46]. In this respect, health professionals are positioned not only as educators but also as policy advocates. They must help individuals interpret labeling while pushing for system‐level clarity.

The data also highlighted the role of narrative and existential meaning in driving lifestyle change. Participants who could link lifestyle decisions to a meaningful story such as protecting family, reclaiming control, or embodying a new identity were better able to commit to change. This affirms Galvin and Todres' [5] argument that lived experience must be integrated into care planning, especially for chronic illness where biomedical facts often overshadow emotional truths.

Healthcare professional interactions varied widely, shaping both motivation and resistance. Participants often experienced care as depersonalised, information‐heavy, and disengaged from their lifeworlds. This is consistent with existing critiques of the over‐medicalised delivery of diabetes care [47, 48]. Where interactions were supportive and dialogical, however, participants described moments of identity validation and renewed agency. These findings call for a shift toward relational, dialogic care that co‐constructs goals and values with patients [7, 49].

Digital resources also emerged as a double‐edged sword offering instant peer support but sometimes amplifying confusion and fear. Healthcare professionals must help individuals critically navigate this space, integrating useful online content with personalised dialogue in clinical settings [50, 51].

Participants' narratives also revealed how prevailing societal and clinical narratives around T2D can be deeply stigmatizing. Several individuals described healthcare encounters where their condition was framed solely in terms of personal responsibility, often linked to weight or lifestyle choices. This kind of discourse, whether implied or explicit, reinforced a sense of shame and blame, and in some cases, led to disengagement from support services. Framing T2D as a “lifestyle illness” not only oversimplifies its complex aetiology, which includes genetic, psychosocial, and environmental contributors, but also risks alienating patients and perpetuating stigma [4, 34]. The international consensus statement on diabetes stigma explains that such narratives foster internalized shame and reduce self‐efficacy while decreasing the usage of healthcare services [4]. Similarly, the NHS Language Matters framework from 2018 highlights how language either supports or challenges diabetes‐related stigma.

Health professionals require support to implement language and attitudes that recognise different T2D causes while prioritising empowerment instead of judgement to promote more inclusive person‐centred care. To address stigma, we must understand how structural and social inequalities shape disease outcomes through access to nutritious food, safe physical activity spaces and equitable healthcare services [52, 53]. The results suggest healthcare settings should transform their perspective on diabetes from one that blames patients to one that understands their situation while moving from compliance‐based approaches to collaborative ones.

Additionally, family dynamics influenced outcomes in similarly nuanced ways. Some families supported collective change and reinforced healthy habits, while others undermined individual efforts or denied the chronic nature of diabetes. As previous research has shown, family members can both buffer and amplify distress [3, 24]. Involving families in care planning and education, not just patients, may enhance sustained behavior change.

Taken together, the findings underscore that lifestyle change in the context of T2D and alcohol use is not simply a matter of individual choice or willpower. It is shaped by narrative, relational, systemic, and emotional factors. Health professionals could adopt person‐centred, narrative‐based approaches to care that validate lived experience while also addressing broader structural and relational challenges. The discussion shows that to support real and lasting change, we must move beyond prescriptive advice to understand how lifestyle change is lived, felt, and made meaningful.

## 6 Limitations

One possible limitation of the study is that all of the participants were recruited from support groups in the north of England, limiting the geographical range of the data. Although qualitative research does not seek to produce statistically representative samples, this is a sampling limitation that does restrict the transferability of the study to other contexts. On the other hand, the sampling strategy was selected with the aim of purposively selecting people whose experiences would be illuminating for the phenomenon of interest, and other geographical regions and populations could be sampled in future studies.

The use of retrospective accounts is a further possible limitation in that these are subject to recall bias as participants reconstruct the past in light of present‐day experiences and emotions. In hermeneutic qualitative research, however, narrative reconstruction is central to the interpretative work, and thus the memory work itself was part of the object of study.

Finally, the analysis was largely done by one researcher, and this can be a source of bias. Rigour was sought by the researcher's ongoing reflexivity and engagement with negative case analysis, the development of a transparent coding system, and peer review of the analysis by another qualitative researcher with considerable experience in the field. Member checking was not used, in line with methodological critiques ([54, 55]) of the usefulness of this technique for interpretive studies.

## 7 Conclusions

This study explored individuals living with T2D who self‐reported alcohol use at varying levels shared their experiences of navigating lifestyle changes within the context of their everyday lives. The findings underscore that lifestyle change is not simply a matter of individual willpower or behavioral compliance but is embedded in social identities, emotional realities, and cultural norms. It also highlights how food and alcohol practices can pose challenges to lifestyle change for some individuals with T2D, particularly when these practices are closely tied to social roles or coping mechanisms. The participants identified food and alcohol as essential elements of their social belonging and group connections. The process of lowering consumption habits demanded individuals to manage changes in their personal identities while balancing health needs against social pressures. People with established ties to social groups focused on alcohol consumption experience deep emotional and relational challenges when switching to healthier lifestyles.

While many participants faced barriers on their journey to change, they found effective support through new social identities created by joining walking clubs and support groups. Through new social connections participants began to view lifestyle changes as components of constructive life stories instead of personal losses.

These results have a number of important implications. In terms of clinical practice, health care providers may need to pay more attention to the social contexts and practices of alcohol and food consumption. Practitioners can offer counsel that recognizes patients' need for identity and belonging, as opposed to only addressing individual behavior. In terms of education, diabetes care training programs might incorporate material on the relational and cultural aspects of lifestyle change, so that practitioners can better understand how to support patients in this way. In terms of future research, future studies could look at interventions to bolster peer and group‐based support. Longitudinal designs may also shed light on how the process of identity change occurs over time.

## Author Contributions

The author conducted all aspects of this study, including its conception, design, data collection, analysis, interpretation, and manuscript preparation. The work was undertaken as part of the author's PhD studies.

## Ethics Statement

Ethical approval for the study was obtained from the University of Leeds Ethics Committee (SHREC) (Ethics Reference: SHREC/RP/527).

## Conflicts of Interest

The author declares no conflicts of interest.

## Data Availability

The data that support the findings of this study are available on request from the corresponding author. The data are not publicly available due to privacy or ethical restrictions.

## Appendix 1

Interview Schedule

- Introduce myself
- Build rapport (allow the participant and the researcher to connect and feel comfortable)

Action Questions

1. Tell me about your experiences with type 2 diabetes?
  - How have you fitted type 2 diabetes condition with your daily life routine?
  - What is this routine for you? – Could you please give me an example?
2. What were you required to do to deal with type 2 diabetes
  - How would you describe the support received in dealing with type 2 diabetes
3. What would say has changed in your lifestyle after the diagnosis with type 2 diabetes? – Give me an example.
4. How has type 2 diabetes affected the levels of alcohol intake for you? – Give me an example.
  - Can you tell me if you feel that modifying your alcohol patterns affected your social life? And in what ways
5. What kinds of support and resources have been most helpful to you in managing lifestyle modifications?
6. How is the relationship between you and the health professionals developed since the diagnosis and onwards?
7. How is your relationship with colleagues, friends, family, and neighbours?

Knowledge Questions

1. What do you know about type 2 diabetes and alcohol use?
  - Can you expand (prompting)
  - How many units or glasses of alcohol did you drink per day and how many do you drink now (prompting)
2. What do you think of the healthcare support you received in managing lifestyle changes for:
  - type 2 diabetes.
  - moderate alcohol?
3. What challenges have you experienced with the changes with regards to lifestyle? / What do you think are the possible solutions to these challenges?

Personal Philosophy Questions

1. How might being diagnosed with type 2 diabetes have changed you as a person?
  - In what ways (prompting question)/example
2. To what degree (extent) do you feel that you have come to terms with the lifestyle changes required from type 2 diabetes
3. How do you feel about your current lifestyle
4. What are your plans for the future?
5. How do you believe that lifestyle changes will affect these plans?

Anything else you would like to add

Thank the participant for the time and the sharing of information and experiences.

## Appendix B

**Table:**

Text	Coding 1 1st cycle coding	Coding 2 2nd cycle coding What is this about? What does it mean? What is happening? What are the assumptions? (make abstract‐touch test)
I guess the most useful thing has been the Diabetic nurse referring me to the Good to Go online course because obviously, they do mention…well there's a big focus on diet, and they do mention alcohol and that was useful to remind me about the amount and the measures of alcohol because it's very easy to have a big wine glass isn't it so I guess I'm a little bit more conscious about not filling the wine glass quite as much but the other resource I think was obviously the Heal project and obviously going to…being a member of the Diabetes UK. I get their magazine Balance every month, and that's got lots of useful information in it and lots of good recipes on their website which I've been using	(1) The most useful thing has been … referring me to the Good to Go online course… (2) There's a big focus on diet and they do mention alcohol, and that was useful to remind me about the amount and the measures of alcohol because it's very easy to have a big wine glass … so I guess I'm a little bit more conscious about not filling the wine glass quite as much… (3) The other resource I think was obviously the Heal project and obviously going to…being a member of the Diabetes UK. I get their magazine Balance every month, and that's got lots of useful information in it and lots of good recipes on their website which I've been using	Internet and volunteer support groups support in altering alcohol and dietary habits (InCL6C1)

In the above coding example, the code “Internet and volunteer support groups support in altering alcohol and dietary habits (InCL6C1)” was grouped with other similar or corresponding codes (see table below) to create a single subtheme.

**Table:**

Subtheme: The internet input in (mis)shaping identities
Codes Mass media provide mixed messages to support consumption rather healthy lifestyles (InBL3C4) The temptations as expressed in current consumerist society impede healthy lifestyles and changes for individuals with type 2 diabetes (InBL18C1) Social media and volunteer support groups provide partial support in altering alcohol and dietary habits (InCL6C1) Greater social awareness of type2 diabetes would enable a more conducive environment for alcohol reduction in individuals with type 2 diabetes (InDL3C1) The use of social media as a means for sharing information (InHL18C1) Self‐educated about diabetes from the internet (InJL10C2) Complimentary to support groups are online social forums that provide extensive and immediate information (InLL4C1) Friends, internet and support groups are the major sources of information (InJL1C3)

The grouping of subthemes together created more abstract conceptual ideas and explanations that were running throughout the data. These constructs are the themes of the study. In the above example, the subtheme “The internet input in (mis)shaping identities” was grouped with other conceptually similar subthemes (see table below) to create a single theme.

**Table:**

Theme: Influences of collective determinants on lifestyle changes
Subthemes Group membership and identity construction Marketing practices of the consumer industry Structural and societal influences on lifestyle choices
